# Promoting Third Graders’ Executive Functions and Literacy: A Pilot Study Examining the Benefits of Mindfulness vs. Relaxation Training

**DOI:** 10.3389/fpsyg.2021.643794

**Published:** 2021-05-20

**Authors:** Carolina Cordeiro, Sofia Magalhães, Renata Rocha, Ana Mesquita, Thierry Olive, São Luís Castro, Teresa Limpo

**Affiliations:** ^1^Faculty of Psychology and Education Sciences, University of Porto, Porto, Portugal; ^2^Centre National de la Recherche Scientifique and University of Poitiers, Poitiers, France

**Keywords:** mindfulness, relaxation, children, executive functions, literacy achievement

## Abstract

Research suggested that developing mindfulness skills in children improves proximal outcomes, such as attention and executive functions, as well as distal outcomes, such as academic achievement. Despite empirical evidence supporting this claim, research on the benefits of mindfulness training in child populations is scarce, with some mixed findings in the field. Here, we aimed to fill in this gap, by examining the effects of a mindfulness training on third graders’ proximal and distal outcomes, namely, attention and executive functions (viz., inhibitory control, working memory, and cognitive flexibility) as well as literacy-related achievement (viz., handwriting fluency, text quality, Portuguese grades). These outcomes were measured with behavioral tasks and teacher ratings. Sixty-six Portuguese children were randomly allocated to an experimental group receiving mindfulness training (*n* = 29) or an active control group receiving relaxation training (*n* = 37). Both training programs were implemented by psychologists in two 30-min weekly sessions for 8 weeks. All students were assessed before and after the interventions. Three main findings are noteworthy: (a) mindfulness training enhanced teacher-rated cognitive flexibility and a performance-based composite score of executive functions among children with higher pretest scores; (b) relaxation training improved performance-based cognitive flexibility and the composite score of executive functions among children with lower pretest scores; (c) children receiving mindfulness training had higher handwriting fluency and better grades in Portuguese than those receiving relaxation training. These findings provide preliminary evidence on the benefits of mindfulness training in educational settings and highlight the moderating role of baseline performance on those benefits.

## Introduction

Mindfulness skills enable individuals to focus on the present moment with a non-judgmental and acceptance attitude toward the experience they are currently living (Kabat-Zinn, 2003; Hooker and Fodor, 2008). It has been suggested that developing these skills in children improves proximal outcomes, such as attention and executive functions (EFs) (Mak et al., 2017; Maynard et al., 2017; Dunning et al., 2019), as well as distal outcomes, such as academic achievement (Maynard et al., 2017). Despite the empirical evidence supporting this claim, research on the benefits of mindfulness training in child populations is still scarce, with some mixed findings in the field. In this study, we aimed to fill in this gap, by examining the effects of a mindfulness-based intervention on third graders’ proximal and distal outcomes, namely, attention and EFs as well as literacy-related achievement, measured through both behavioral tasks and teacher ratings.

Executive functions enable individuals to successfully engage in independent and purposeful behavior. According to Diamond (2013), EFs include working memory, inhibitory control, and cognitive flexibility. Together with attention, these skills are critical to achieve success in school (Mulder and Cragg, 2014), and considered proximal outcomes of mindfulness-based interventions (Lyons and DeLange, 2016; Takacs and Kassai, 2019). Some studies showed mindfulness benefits on global executive functioning measured with either performance-based tasks (Parker et al., 2014) or teacher/parents’ ratings (Flook et al., 2010). Other child studies found a positive impact of mindfulness-based interventions in specific components of EFs, such as enhanced selective attention skills (Napoli et al., 2005; Felver et al., 2014) and higher working memory capacity (Ricarte et al., 2015), as well as improved inhibitory control and cognitive flexibility (Janz et al., 2019). Still, findings are not consensual, with other studies reporting no mindfulness benefits on attention or EFs (Mak et al., 2017), including in adults (Semple, 2010; Josefsson et al., 2014).

Though to a less extent, the effects of mindfulness have also been explored in distal outcomes, such as academic achievement (Maynard et al., 2017). Experimental research showed that this kind of training improved children’s reading and science grades (Bakosh et al., 2015) and math performance (Schonert-Reichl et al., 2015). Correlational research also showed that higher mindfulness skills were correlated with grade point average and standardized tests of math and literacy (Caballero et al., 2019). No research has, however, linked mindfulness practice with children’s ability to produce good writing, which is a fundamental academic skill across all school subjects.

Typically, mindfulness-based interventions target the following key components (Nhat Han, 1990): awareness of the body (i.e., be aware of the body and sensations at the present moment), awareness of feelings (i.e., express appropriately emotions and cultivate and deal with positive and negative emotions), awareness of thoughts (i.e., understand how thoughts work); and awareness of relationships (i.e., understand other people’s behaviors and communicate with others). The most common activities used to train these components are meditation-based activities (e.g., breathing techniques, sitting meditation, attention to internal and external sensations, body scan) and exercises to identify and deal with negative emotions and thoughts (e.g., perspective taking exercises). Together, these features of mindfulness-based interventions are thought to improve proximal and distal outcomes through several cognitive mechanisms (Schonert-Reichl and Roeser, 2016). Meditation activities may improve students’ attention by requiring them to focus on relevant stimulus in a sustained manner as well as enhance EFs by training their abilities to concentrate on the information available on the present moment (working memory), to inhibit distracting information to the task at hands (inhibitory control), and to adapt to new rules or to adjust their approach to a problem (cognitive flexibility).

The mindfulness-related improvements on attention and EFs are expected to generalize to academic achievement (Maynard et al., 2017). Past research already showed that these cognitive skills are related to teacher-rated literacy grades (Magalhães et al., 2020) as well as to specific, writing-related indicators of literacy abilities (Cordeiro et al., 2019). In primary grades, two of these indicators are transcription, which is the externalization of language into written text through the basic processes of handwriting and spelling; and written composition, that is, the production of texts with good and coherently organized ideas, conveyed in well drafted sentences with rich vocabulary. Indeed, attention and EFs has been proposed as central processes in influential cognitive models of writing (Graham, 2018). It thus seems plausible to hypothesize that by promoting attention and EFs, mindfulness interventions may also improve these writing-related skills.

## Present Study

As a recent topic of inquiry, research supporting mindfulness-based intervention effects on children’s cognitive and academic skills is reduced and controverse. Also, some previous studies showed methodological frailties, involving a lack of randomized designs and active control groups (Mak et al., 2017; Dunning et al., 2019). The present study aimed to increase current knowledge on the effects of mindfulness training in Portuguese third graders, using a randomized control trial design and an active control group receiving relaxation training. This type of training is a powerful and commonly used comparison treatment to test the effects of mindfulness over mere relaxation techniques (Jain et al., 2007; Manocha et al., 2011). We examined the impact of an 8-h mindfulness intervention on the proximal outcomes of attention and EFs (measured with cognitive tasks and teacher ratings), and on the distal outcomes of literacy-related achievement (measured via writing tasks and teacher-assigned Portuguese grades). Based on the previously surveyed findings, we hypothesized that, after the interventions, students in the experimental group (mindfulness) would surpass their peers in the active control group (relaxation) in terms of attention and EFs (Mak et al., 2017; Dunning et al., 2019), as well as literacy-related skills (Maynard et al., 2017). Together, these findings may contribute to a better understanding of how mindfulness can be used to enhance cognitive processes and academic skills critical for children’s success in school and in life.

## Methods

### Participants and Experimental Design

Eighty 3rd graders from a School in the North of Portugal were authorized by their legal guardians and agreed to participate in the study, which was approved by the Ethics Committee of the Faculty of Psychology and Education Sciences of the University of Porto. Within each class and using random.org, all children were randomly assigned to a mindfulness program (experimental group), or to a relaxation program (active control group). Although all children participated in an intervention for ethical reasons, to define the data-analytic sample we excluded children with special education needs (*n* = 7) and non-native speakers of Portuguese (*n* = 2). From the eligible sample of 71 students, five were dropped due to non-compliance with assessment procedures (e.g., did not finish the task or did not execute it properly). The final data-analytic sample included 66 students (respectively, 29 and 37 in the mindfulness and relaxation conditions).

Table 1 presents a characterization of both groups, which did not differ in terms of age, *t*(64) = 1.36, *p* = 0.17, gender χ^2^(1) = 0.01, *p* = 0.56, and non-verbal intelligence, *t*(64) = −1.52, *p* = 0.23. However, despite the randomization, the mothers of children in the mindfulness group were found to have higher educational levels than those in the relaxation group, *t*(64) = −2.26, *p* = 0.04. This difference was controlled for in all statistical analyses (see section “Results”).

**TABLE 1:** Participants characteristics by intervention group.

	Mindfulness (*n* = 29)	Relaxation (*n* = 37)
Number of girls	13	17
**Age (in years)**		
M (*SD*)	8.22 (0.30)	8.33 (0.32)
Min-Max	7.73–8.79	7.80–9.02
**Raven**		
M (*SD*)	25.55 (5.90)	23.51 (5.03)
Min-Max	14–34	12–35
**Mother educational level (*n*)**		
Grade 4	3	5
Grade 9	15	29
Grade 12	7	1
University degree	4	2

### Educational Setting

This study took place in a school labeled as “territory of priority educational intervention.” This is a Portuguese governmental initiative to prevent and reduce early school dropout and absenteeism in economic and social disadvantaged areas, where violence, indiscipline, dropout, and school failure are common problems.

### Intervention Programs

Both interventions were implemented in groups of 7–8 students during 16 30-min sessions, delivered twice a week by two trained psychologists (each psychologist worked with half of the mindfulness and relaxation groups). Detailed descriptions of the activities implemented in each intervention are provided in Supplementary Tables 1, 2.

**TABLE 2:** Descriptive statistics for all measures at both testing sessions by intervention group.

	Pretest				Posttest							
		
	Mindfulness		Relaxation		Mindfulness				Relaxation			
				
	*M*	*SD*	*M*	*SD*	*M*	*SD*	*M*_*adj*_	*SE*	*M*	*SD*	*M*_*adj*_	*SE*
**Proximal outcomes – cognitive tasks**												
Attention	6.93	1.71	7.26	3.77	10.88	2.16	11.03	0.45	11.21	4.31	11.10	0.40
Working memory	4.91	1.10	4.55	0.87	5.52	1.29	5.33	0.16	5.20	1.05	5.36	0.18
Inhibition	9.28	3.12	8.95	2.70	11.45	3.62	11.17	0.55	10.57	2.72	10.78	0.49
Cognitive flexibility	37.00	7.95	35.00	8.98	38.21	8.83	37.78	1.43	38.16	7.16	38.49	1.26
Composite score	0.13	0.71	−0.11	0.65	0.10	0.76	−0.003	0.09	−0.08	0.52	<−0.001	0.08
**Proximal outcomes – teacher based**												
Attention	3.49	0.88	3.53	0.82	3.75	0.92	3.77	0.09	3.70	0.90	3.69	0.09
Working memory	3.85	0.87	3.91	0.85	4.10	0.91	4.12	0.08	3.99	0.93	3.97	0.08
Inhibition	3.83	0.72	3.92	0.71	3.91	0.79	3.70	0.07	3.97	0.75	3.78	0.07
Cognitive flexibility	3.34	0.67	3.22	0.73	3.48	0.75	3.42	0.10	3.14	0.66	3.20	0.09
Composite score	3.67	0.70	3.68	0.68	3.83	0.76	3.83	0.07	3.70	0.71	3.71	0.06
**Distal outcomes – writing tasks**												
Handwriting fluency	9.28	4.07	10.16	4.17	14.03	5.34	14.37	0.85	11.84	4.66	11.58	0.75
Spelling accuracy	5.66	2.70	4.32	2.74	6.66	2.42	6.11	0.32	5.78	2.75	6.21	0.28
Text quality	2.50	0.74	2.15	0.82	2.78	0.97	2.44	0.20	2.85	1.01	2.79	0.17
**Distal outcomes – teacher based**												
Portuguese grades	4.24	0.83	3.81	0.74	4.10	0.72	3.97	0.92	3.59	0.64	3.70	0.81

**M*_*adj*_ corresponds to means adjusted for pretest scores and mothers’ educational level. Sample size for EF teacher reported measures is 41 (20 for mindfulness group and 21 for relaxation).*

#### Mindfulness

Grounded on past studies (e.g., Viafora et al., 2015; Thomas and Atkinson, 2016) and on a literature review, in which we identified the most effective mindfulness activities (Magalhães et al., 2019), we developed a program organized into three components: calming the mind (viz., listen to a resonance instrument and focus attention on breath); consciously attending to internal and external stimuli (viz., observe sensations, emotions, and thoughts); and dealing with negative emotions and thoughts (viz., reflect on negative emotions and thoughts, with and attitude of acceptance and compassion). Before conducting the present study, the intervention was pilot tested with a different sample of third graders to rectify procedures and activities.

#### Relaxation

This program was based on Koeppen (1974) and included activities aimed to promote progressive muscle relaxation of seven muscle groups: hands and arms; chin and mouth; face and nose; stomach; arms and shoulders; neck and shoulders; and feet and legs.

#### Treatment Fidelity

To ensure that both interventions were delivered as intended we applied four procedures. First, instructors participated in an 8-week pre-intervention course, where they were introduced to the theory and practice of mindfulness. At the end of the course, they received the instructional manuals and discussed intervention procedures. Second, during the interventions, instructors had weekly meetings to discuss previous sessions and prepare the next ones. Though rare, deviations from instructional plans were solved in the subsequent sessions. Third, at the end of each session, the instructor completed a checklist with all lessons’ steps implemented. Excepting one session in a relaxation group, in which one out of nine steps was not conducted, 100% of the steps were completed in all other groups. Fourth, a research assistant observed three sessions for all mindfulness and relaxation groups and filled in the same checklists as the instructors. Results showed full compliance with the manuals in both conditions.

### Testing Sessions

Before and after the interventions, all students participated in a 30-min group session and another 30-min individual session. In the group session, students performed the writing tasks (opinion essay writing, alphabet task, spelling through dictation). In the individual session, students were asked to perform the attention and EFs tasks. Within each session, the order of tasks was held constant. All tasks were administered by duly trained psychologists with an equivalent amount of pre-training and experience. Pretest assessments were conducted by the instructors, whereas posttest assessments were implemented by other psychologists. Before and after the interventions, teachers provided students literacy-related grades and completed a set of EFs rating scales for each child (unfortunately, we only received complete pretest and posttest data of 41 children). All psychologists and teachers involved in the assessments were blind to students’ condition.

### Measures

In what follows, we summarize the assessment measures used. A detailed description of these, including information confirming their good psychometric properties, is provided in Supplementary Table 3.

#### Non-verbal Intelligence

To assure groups equivalence, we used the Raven’s colored progressive matrices (Simões, 2000; Raven et al., 2004).

#### Proximal Outcomes – Cognitive Tasks

We measured attention with the Cancelation task from the Coimbra Neuropsychological Assessment Battery (BANC; Simões et al., 2016), and the three core EFs of working memory, inhibitory control, and cognitive flexibility with the following tasks, respectively: Digit span task from the Wechsler Intelligence and Scale for Children-III (Simões et al., 2003); Inhibition subtest of the NEPSY-II, A Development Neuropsychological Assessment (Korkman et al., 2007); and the semantic fluency task from BANC (Simões et al., 2016). A composite score representing these three core domains of EF (Diamond and Ling, 2016) was computed by averaging the standardized scores of the working memory, inhibitory control, and cognitive flexibility both at pretest and posttest.

#### Proximal Outcomes – Teacher Based

We used the four scales of the Comprehensive Executive Function Inventory (Naglieri and Goldstein, 2013) that targeted the same dimensions measured with behavioral tasks: attention, working memory, inhibitory control, and cognitive flexibility. As before, working memory, inhibitory control, and cognitive flexibility scores were averaged to achieve a composite score both at pretest and posttest. Preliminary validity evidence on this instrument was provided by Carvalho (2020).

#### Distal Outcomes – Writing Tasks

We measured transcription abilities through student’s handwriting fluency skills in an alphabet task (Limpo and Alves, 2018), and spelling skills through a dictation task (Magalhães et al., 2020). To measure students’ written composition skills, we asked two trained judges to rate the quality of their opinion essays, using a holistic scale ranging from 1 (*low quality*) to 7 (*high quality*) (based on Limpo and Alves, 2018).

#### Distal Outcomes – Teacher Based

As an indicator of students’ achievement in the literacy domain we gathered students school grades for the Portuguese subject at the end of Grade 2 and at the end of the first period of Grade 3 (these were the most recent grades, respectively, before and after the interventions).

## Results

Preliminary analysis showed no distributional problems in either group, as the absolute values of skewness and kurtosis for all pretest and posttest variables were below |1.25| and |2.20|, respectively (Kline, 2005). To test for differences between mindfulness and relaxation effects on proximal and distal outcomes, we conducted one-way analyses of co-variance (ANCOVAs). Given the unequal group size, we used the Type III Sum-of-Squares method (Tabachnick and Fidell, 2007). As covariates, we introduced the pretest score of the dependent variable under analysis along with the educational level of children’s mothers (see Table 2 for descriptive statistics). Although we used a true random design and there were no pretest differences in proximal and distal outcomes (all *p*s > 0.07), controlling for pretest scores increases statistical power by reducing error variance. Based on Tabachnick and Fidell (2007), the educational level of children’s mothers was also introduced as a covariate because the mindfulness group included more educated mothers than the relaxation group (see section “Participants and Experimental Design”). Not controlling for this difference could be problematic as prior research showed an association between mothers’ educational level and our dependent variables, namely, children’s EFs (e.g., Ardila et al., 2005), writing performance (e.g., Rindermann et al., 2011), and academic achievement (e.g., Idris et al., 2020). For all analyses, the assumption of homogeneity of variances was met. Moreover, before examining condition effects on dependent variables, after controlling for the respective pretest score and mothers’ educational level, we examined the homogeneity of the regression slopes across groups. This is a central assumption of ANCOVA, whose violation impedes its execution. To test it, we first introduced the two two-way interactions between condition and pretest score and between condition and mothers’ educational level, and then we added the three-way interaction. Significant interactions would mean that the assumption of homogeneous regression slopes was not met, or in other words, that condition effects were moderated by one or two co-variates. In these cases, we used the Johnson-Neyman (J-N) procedure to determine the regions of significance for the Condition × Covariate/s interaction (Aiken and West, 1991). Regions of significance define the levels of the covariate/s at which there is a significant condition effect (Bauer and Curran, 2005). This procedure was implemented with the PROCESS macro for SPSS, version 3 (Hayes, 2018). In what follows, we present significant findings involving condition effects, using an alpha level of 0.05. Adjustments for multiple comparison were not made, as these would be very conservative, particularly given the small sample size (Perneger, 1998). Complete ANCOVAs results are presented in Supplementary Table 4.

### Effects on Proximal Outcomes – Cognitive Tasks

The assumption of homogeneous regression slopes was met for attention, working memory, and inhibition scores. However, ANCOVA results showed no evidence of condition effects. For cognitive flexibility, we found an interaction between condition, pretest scores, and mother’s educational level, *F*(2,58) = 6.40, *p* = 0.003, η^2^_*p*_ = 0.18, indicative of a violation of the assumption of homogeneous regression slopes. The J-N procedure was then used. Results showed that for children whose mothers finished Grade 9 (67% of the sample), the relaxation training resulted in higher cognitive flexibility than the mindfulness training among children with lower cognitive flexibility at pretest, *b* = −6.75, *t* = −2.02, *p* = 0.05 (scores equal to or below 29, 18% of the sample with mothers with Grade 9 completed).

The J-N procedure was also used to examine condition effects on the composite score of EFs, because there was a significant interaction between condition and pretest scores, *F*(1,60) = 9.11, *p* = 0.004, η^2^_*p*_ = 0.13. Result showed that, on the one hand, for children with lower composite scores at pretest (equal to or below −0.58; 17% of the sample), the relaxation training was more effective than the mindfulness training, *b* = −0.29, *t* = −2.00, *p* = 0.05. On the other hand, for children with higher composite scores at pretest (equal to or above 0.60; 18% of the sample), the mindfulness training resulted in higher composite scores than the relaxation training, *b* = 0.30, *t* = 2.00, *p* = 0.05.

### Effects on Proximal Outcomes – Teacher Based

The assumption of homogeneous regression slopes was met for attention, working memory, inhibition, and EFs composite scores. However, ANCOVA results showed no evidence of condition effects. For cognitive flexibility, results revealed an interaction between condition and pretest scores, *F*(1,35) = 4.85, *p* = 0.03, η^2^_*p*_ = 0.12, meaning that the assumption of homogeneous regression slopes was violated. The J-N procedure showed that for children with pretest flexibility scores equal to or above 3.42 (41% of the sample), the mindfulness training resulted in better cognitive flexibility than the relaxation training, *b* = 0.26, *t* = 2.03, *p* = 0.05.

### Effects on Distal Outcomes – Writing Tasks and Teacher Based

For handwriting fluency, spelling, text quality, and Portuguese grades, the assumption of homogeneous regression slopes was met. ANCOVA results showed that there were no effects of condition on spelling and text quality. However, results showed differences between condition on handwriting fluency, *F*(1,62) = 5.85, *p* = 0.02, η^2^_*p*_ = 0.09, and Portuguese grades, *F*(1,62) = 4.39, *p* = 0.04, η^2^_*p*_ = 0.07. At posttest, the mindfulness group showed greater handwriting fluency and better Portuguese grades than the relaxation group.

## Discussion

We conducted a randomized active-controlled study to test the effects of an 8-h mindfulness-based intervention on proximal and distal outcomes in a sample of Portuguese third graders. In what follows, we discussed the results, which partially confirmed our hypotheses.

### Effects on the Proximal Outcomes of Attention and EFs

Contrary to our expectations, we did not find group differences on measures of attention, inhibitory control, and working memory. These findings contrast with previous research (Felver et al., 2014; Ricarte et al., 2015; Schonert-Reichl et al., 2015), even though a recent meta-analysis including an independent effect size calculation reported that eight out of 13 studies showed no improvements in attention and EFs after mindfulness training (Mak et al., 2017). The lack of findings can be explained by methodological features of the studies, such as the measures used, which seem to have a key role in capturing mindfulness effects. Probably, there was a mismatch between the measures used to assess attention, inhibitory control, and working memory and the meditation-based activities used to train these skills (Zenner et al., 2014). For example, some of our tasks asked for time-limited responses to external targets (e.g., signs), whereas the mindfulness training targeted children’s ability to be aware of current internal objects (e.g., sensations, emotions, and thoughts). As previously noted (Josefsson et al., 2014), some similarity between mindfulness and relaxation training (e.g., focus on the body, induction of calmness) may have also contributed to the absence of condition effects (e.g., Jain et al., 2007; Luberto et al., 2020).

Partially confirming our hypotheses, there were condition effects on performance-based and teacher-rated cognitive flexibility and on a performance-based composite score of EFs, which were moderated by participants characteristics. Overall, mindfulness training worked better for those with higher EFs at pretest, whereas the relaxation training benefited more those with lower EFs at pretest. These findings are in contrast with those of Flook et al. (2010), who showed that school-based program of mindful awareness practices resulted in stronger improvements in EFs among children with EF difficulties (see also Flook et al., 2015). In addition to the use of a less stringent control group (silent reading), there was a key different between their study and ours: The mindfulness program tested here not only included mediation exercises targeting different levels of awareness as the one tested by Flook et al. (2010), but it also included several reflective practices focused on thoughts and emotions. The amount of reflection required by the mindfulness training, totally absent in the relaxation training, may explain the differential moderating role of EF. The importance of EFs in learning is not new (Shaul and Schwartz, 2014). This set of skills allow learners to sit still in their place and pay attention to the teacher, as well as to acquire new material and integrate it on what was already learned (Meuwissen and Zelazo, 2014). It is thus likely that higher EFs at baseline may facilitate learning during highly reflective programs engaging several cognitive skills (for a review on theoretical and empirical evidence supporting this claim see Zelazo, 2015). Previous research already showed evidence of greater benefits from mindfulness-based training for older adults with higher cognitive resources (Whitmoyer et al., 2020), or adolescents with lower attentional problems (Fung et al., 2019). Contrary to mindfulness training, the relaxation techniques practiced by the control group relied little on children’s cognitive skills (McCallie et al., 2006). Throughout the intervention, they were only asked to follow explicit, simple, and clear rules to contract body parts, without any kind of reflection or conceptual change. The reduced cognitive and emotional demands of progressive muscle relaxation may turn this type of training more suited for those with cognitive vulnerabilities (Luberto et al., 2020).

It should, however, be noted that, before drawing conclusions about these findings, mindfulness research is still in its infancy. Moreover, studies examining the moderating role of baseline EF status in mindfulness training are scarce, with mixed findings. Indeed, an open question in the field of cognitive training is whether it works better for those with higher or lower cognitive skills at baseline (i.e., magnification vs. compensation effects; for a review on this see Titz and Karbach, 2014). Future research should delve into the differential effects of mindfulness and relaxation, as both types of interventions seem to be beneficial, depending on children’s characteristics. From an applied viewpoint, this means that psychologists and teachers may be able to select one or another intervention in order to meet the unique characteristics of children, with maximized chances of success. To understand the mechanisms and conditions through which mindfulness and relaxation training may improve children’s EFs seem therefore an important future research avenue.

### Effects on the Distal Outcomes of Literacy-Related Skills

Concerning writing tasks, though there were no mindfulness effects on spelling and text quality, children receiving mindfulness training showed greater handwriting fluency than their peers. Likely, this training may have reduced distracting thoughts, allowing them to allocate more attentional and cognitive resources to the task (Mrazek et al., 2013). Still, such hypothesis does not explain why the enhanced resources increased handwriting fluency but not spelling and written composition. Despite related, handwriting, spelling, and writing rely on different processes (Graham, 2018), which seem to be differentially affected by mindfulness training. More research is needed to unravel the effects of mindfulness interventions on specific writing processes.

The most relevant finding of the present study was that children in the mindfulness intervention achieved better Portuguese grades than their peers. A handful of past works already showed the benefits of mindfulness on key domains of academic achievement indexed via teacher-reported grades (Bakosh et al., 2015). In addition to attention and EFs (Mak et al., 2017; Dunning et al., 2019), it has been suggested that mindfulness activities help children to cultivate a range of skills such as emotional regulation, stress management, awareness, and resilience (e.g., Zenner et al., 2014), that may explain these distal effects on teacher-reported grades. The role of those variables as mediators of mindfulness-based intervention effects on school achievement is certainly a central area of inquiry to unravel the mechanisms through which mindfulness interventions work.

### Limitations and Future Directions

When interpreting current findings at least five limitations should be kept in mind. First, the sample of this study belongs to a particular educational context, prone to school absence, violence, and failure. Thus, results may not generalize to other contexts, which may require further research attention. Second, we conducted a pilot study with a small sample size, particularly for the teacher-rated EFs, which may have weakened its statistical power and increase the likelihood of Type II errors (Abraham and Russell, 2008). The design of subsequent studies should include *a priori* power analysis to estimate sample size. Third, despite the randomization procedure, the mindfulness group included children with more literate mothers than the relaxation group. This difference was statistically controlled, but more tests of the effects of the current program are needed. It should be noted that the research questions in this study were not directly related to determine the moderating role of mothers’ educational level. Thus, results involving it should be read carefully. Follow-up studies targeting the role of children’ background in mindfulness interventions should complement mothers’ educational level with other information, including from the father. Fourth, for 36% of the sample, we had no information on teacher-rated attention and EFs skills, as teachers did not return the questionnaires. Results concerning those measures should therefore be read carefully and replicated in the future. Finally, the experimental and control groups shared a focus on the body that, though allowing a stringent test to the effects of mindfulness-specific features, may explain the limited condition differences here observed. In addition to an active control group, future studies should include passive control groups (e.g., waiting lists) for a better understanding of mindfulness effects above and beyond related-forms of treatment or no treatment.

### Conclusion

This study showed that a mindfulness intervention as short as 8 h improved third graders performance-based global EFs and teacher-rated cognitive flexibility among those with higher EFs at pretest as well as handwriting fluency and Portuguese grades among all children. Moreover, the same amount of relaxation practice improved children’s performance-based global EFs and cognitive flexibility among those with lower EFs at pretest. Though more research is needed into the proximal and distal outcomes of mindfulness (and relaxation) training, along with the key variables moderating and mediating those effects, the current study’s findings are encouraging with regard to the added value of incorporating mindfulness into primary school.

## Data Availability Statement

The raw data supporting the conclusions of this article will be made available by the authors, without undue reservation.

## Ethics Statement

The studies involving human participants were reviewed and approved by the Faculty of Psychology and Education Sciences of the University of Porto. Written informed consent to participate in this study was provided by the participants’ legal guardian/next of kin.

## Author Contributions

TL, SC, and TO designed the study, supervised all other tasks, and reviewed the manuscript. SM designed the intervention programs and trained instructors. CC, RR, and AM implemented the programs and collected and coded the data. TL and CC analyzed and interpreted the data. CC wrote the first version of the manuscript. All authors approved the final version.

## Conflict of Interest

The authors declare that the research was conducted in the absence of any commercial or financial relationships that could be construed as a potential conflict of interest.
