# Supplementary material for: Promoting Third Graders’ Executive Functions and Literacy: A Pilot Study Examining the Benefits of Mindfulness vs. Relaxation Training
Source: Front Psychol. 2021 May 20;12:643794. doi: 10.3389/fpsyg.2021.643794 (PMC8172966; doi:10.3389/fpsyg.2021.643794)
Supplement: Supplementary file 1 [file Table_1.DOCX]

Supplementary Material

# Supplementary Table S1: Description of the Activities Carried out in the Mindfulness Program.

|  | Activities | Description |
| --- | --- | --- |
| Mindfulness of breathing | | Children were instructed and guided to focus their attention on breathing sensations |
| Mindfulness of sounds | | Children were instructed and guided to focus their attention on one sound (e.g., Tibetan bowl) |
| Mindful eating | | Children were instructed and guided to focus their attention on sensory experiences of a meal (smell, sound, touch, taste, and aspect) |
| Mindfulness of movements | | Children were instructed and guided to focus their attention on body sensations while standing, walking, stretching or playing |
| Body scan | | Children were instructed and guided to slowly direct their attention to different parts of their bodies, from toes to head |
| Mindfulness of emotions | | Children were instructed and guided to observe and identify the emotions in the present moment, including reflecting on and dealing with the most unpleasant emotions (e.g., sadness and anger) |
| Mindfulness of thoughts | | Children were instructed and guided to observe and identify the thoughts in the present moment, including reflecting on and dealing with the most unpleasant thoughts (e.g., I cannot do it) |
| Perspective taking | | Role-play of situations related to children’s daily lives to consolidate the knowledge acquired in the sessions |
| Reflection | | Reflect about the lessons learned and generalize to daily live (e.g., discussion of possible ways to apply knowledge in routine situations) |

# Supplementary Table S2: Description of the Activities Carried out in the Relaxation Program.

| Activities | Description |
| --- | --- |
| Hands and arms | Children were instructed and guided to contract and then relax twice as long their hands and arms (e.g., pretend they are squeezing oranges) |
| Chin and mouth | Children were instructed and guided to contract and then relax twice as long their chin and mouth (e.g., pretend they are chewing a bubble-gum |
| Face and nose | Children were instructed and guided to contract and then relax twice as long their face and nose (e.g., pretend a pesky fly landed on their nose) |
| Stomach | Children were instructed and guided to contract and then relax twice as long their stomach (e.g., pretend they have to protect them from a baby elephant) |
| Arms and shoulders | Children were instructed and guided to contract and then relax twice as long their arms and shoulders (e.g., pretend they are a lazy cat and they want to stretch) |
| Neck and shoulders | Children were instructed and guided to contract and then relax twice as long their neck and shoulders (e.g., pretend they are a turtle and they sense danger) |
| Feet and legs | Children were instructed and guided to contract and then relax twice as long their feet and legs (e.g. pretend they are standing barefoot in a mud puddle) |

# Supplementary Table S3: Description of the Measures.

*Note.* All measures described below were collected before and after the intervention programs, except the Raven’s colored progressive matrices, only administered before the interventions.

| Construct | Measure | Description | Final score | Validity/Reliability |
| --- | --- | --- | --- | --- |
| Control measure | | | | |
| Non-verbal intelligence | Raven’s colored progressive matrices (Raven et al., 2004; Simões, 2000) | Children need to identify the missing element of a given pattern among six options. It includes three sets of 12 items. | Sum of correct answers | Good internal consistency (.65 < $\alpha$ < .88) |
| Proximal outcomes | | | | |
| Attention | Cancellation task from BANC (Simões et al., 2016). | Children are given a sheet with squares organized in lines and are asked to cross out the squares that match a previously presented model. | Formula that considers the squares correctly crossed, omitted, and incorrectly crossed. | Good stability coefficient through test-retest (*r =* .61) and validity indices (.24 < *r <* .58). |
| Working memory | Digit Span task from WISC-III (Simões et al., 2003). | Children are asked to recall sequences of numbers with increasing length in forward and backward order. | Average of sequences completed in forward and backward order. | Good stability coefficient (*r =* .80). |
| Inhibitory control | Inhibition subtest of the NEPSY-II (Korkman et al., 2007). | Children are given a sheet depicting black and white shapes (Part I) or arrows (Part II) and are asked to say the opposite form (i.e., saying square when circle and vice-versa) or arrow direction (i.e., saying up when pointing down and vice-versa). | Combined score considering completion time (max. 240s) and errors. | Good test-retest reliability (*r =* .81) and excellent internal consistency ($\alpha$ = .92). |
| Cognitive flexibility | Semantic fluency task from BANC (Simões et al., 2016). | For each category (animal, names, and food), children were asked to produce the maximum number of examples during 60 s. In each category, the examiner gave two examples of possible words. | Sum of correct words in the three categories. | Good stability coefficient (*r* = .79). |
| Proximal outcomes – teacher based | | | | |
| Same domains measured with behavioral tasks | Comprehensive Executive Function Inventory (CEFI; Naglieri & Goldstein, 2013) validated to Portuguese by Carvalho (2020). | Comprehensive behavior rating scale of executive-function strengths and weaknesses, organized into nine dimensions. Only the subscales of attention (12 items), working memory (11 items), inhibitory control (10 items), and cognitive flexibility (7 items) were considered in the present study. | Average of items on each subscale. Answers were given using a Likert-type scale (1 = *never* to 6 = *always*). | Respectively, at prestest/ posttest, Cronbach’s alphas were: .96/.97 for attention, .96/.97 for working memory, .89/.93 for inhibition, and 86/.87 for flexibility. |
| Distal outcomes – writing tasks | | | | |
| Handwriting fluency | Alphabet task(Limpo & Alves, 2018). | Students were asked to write the alphabet in lowercase as quickly as possible, without making mistakes, during 15 seconds. | Number of correct letters written. | A second judge rescored this task for 25% of the students. Interrater reliability, measured by the intraclass correlation coefficient (ICC), was .96 at pretest and 1.00 at posttest. |
| Spelling | Dictation task (Magalhães et al., 2020). | Dictation of 16 isolated words that represented the following complexities of the Portuguese spelling system: complex graphemes, silent letter *h,* contextual effect, position effect, inconsistency, consonantal group, and stress marks. | Number of spelling errors. | A second judge rescored this task for 25% of the students. ICC was .94 at both testing moments. |
| Text quality | Opinion essay (based on Limpo & Alves, 2018). | Children were given 10 min to write their opinion about a topic (pretest: “Do you think there should be more field trips at school?”; posttest: “Do you think teachers should give homework every day?”). To avoid biased judgements, we removed identifying, randomly organized all texts, and and typed the texts correcting for spelling errors. | Two research assistants, blind to study purposes, gave an overall quality judgment ranging from 1 to 7, considering the following factors: ideas quality, organization, sentence structure, and vocabulary. For each text, the quality scores were the average of the two judges. | ICC for average measures was .85 for pretest, and .88 for posttest. |
| Proximal outcomes – teacher based | | | | |
| Literacy-related academic achievement | Portuguese grades | This measure corresponds to the teacher overall evaluation of each student, considering the following curricular domains: oral comprehension, oral expression, literary education, written expression, and explicit knowledge of the language (Ministério da Educação, 2018). This grade is given three times a year, at end of the term. In the present study, we used the grades assigned immediately before and after the intervention. | The qualitative grades assigned by teachers were transformed into a numerical scale ranging from 1 (*lowest score*) and 5 (*highest score*). | In Portuguese primary grades these measures have already been linked with measures of attention and EFs (Magalhães et al., 2020). |

**References**

Carvalho, D. (2020). Validação preliminar de um instrumento para avaliar as funções executivas em crianças portuguesas [Preliminary validation of an instrument to measure executive functions in Portuguese children] [Unpublished master dissertation]. University of Porto

Korkman, M., Kirk, U., & Kemp, S. (2007). *NEPSY -II: Administration manual*. Harcourt Assessment.

Limpo, T., & Alves, R. A. (2018). Tailoring multicomponent writing interventions: Effects of coupling self-regulation and transcription training. *Journal of Learning Disabilities*, *51*(4), 381-398. https://doi.org/10.1177/0022219417708170

Magalhães, S., Mesquita, A., Filipe, M., Veloso, A., Castro, S. L., & Limpo, T. (2020). Spelling performance of Portuguese children: Comparison between grade level, misspelling type, and assessment task. *Frontiers in Psychology*, *11*(547), 1-9. https://doi.org/10.3389/fpsyg.2020.00547

Ministério da Educação. (2018). Aprendizagens essenciais: Articulação com o perfil dos alunos. [Essential learnings: Articulation with the profile of students]. https://www.dge.mec.pt/sites/default/files/Curriculo/Aprendizagens_Essenciais/1_ciclo/portugues_1c_3a_ff.pdf

Naglieri, J. A., & Goldstein, S. (2013). *Comprehensive executive function inventory: Technical manual*. Multi-Health Systems Inc.

Raven, J., Raven, J. C., & Court, J. H. (2004). *Manual for Raven’s Progressive Matrices and Vocabulary Scales*. Harcourt Assessment.

Simões, M. (2000). *Investigações no âmbito da aferição nacional do Teste das Matrizes Progressivas Coloridas de Raven (M.P.C.R.)*. Fundação Calouste Gulbenkian.

Simões, M., Albuquerque, C., Pinho, M., Vilar, M., Pereira, M., Lopes, A., Santos, M., Alberto, I., Lopes, C., Martins, C., & Moura, O. (2016). *Bateria de Avaliação Neuropsicológica de Coimbra*. CEGOC-TEA.

Simões, M., Rocha, A. M., & Ferreira, C. (2003). *WISC-III, Escala de Inteligência de Wechsler para Crianças - 3ª edição*. CEGOC-TEA.

# Supplementary Table S4: Complete ANCOVAs Results.

*Note.* Results are not presented for cognitive flexibility measured through cognitive tasks and teacher ratings as well as for the composite score of EFs tasks because, due to the violation of the assumption of homogeneity of the regression slopes, the Johnson-Neyman (J-N) procedure was used instead of the ANCOVA.

|  |  | Pretest score effect | | |  | Mothers’ educational level effect | | |  | Condition effect | | |
| --- | --- | --- | --- | --- | --- | --- | --- | --- | --- | --- | --- | --- |
|  |  | *F* | *p* | η^2^_p_ |  | *F* | *p* | η^2^_p_ |  | *F* | *p* | η^2^_p_ |
| Proximal outcomes – cognitive tasks | |  |  |  |  |  |  |  |  |  |  |  |
|  | Attention | 80.20 | < .001 | .56 |  | 0.03 | .87 | < .001 |  | 0.01 | .91 | < .001 |
|  | Working memory | 27.90 | < .001 | .31 |  | 0.31 | .58 | .005 |  | 0.02 | .89 | < .001 |
|  | Inhibition | 8.52 | .01 | .12 |  | 3.27 | .08 | .05 |  | 0.27 | .61 | .004 |
|  | Cognitive flexibility | - | - | - |  | - | - | - |  | - | - | - |
|  | Composite score | - | - | - |  | - | - | - |  | - | - | - |
| Proximal outcomes – teacher based | |  |  |  |  |  |  |  |  |  |  |  |
|  | Attention | 168.16 | < .001 | .82 |  | 0.14 | .71 | .004 |  | 0.41 | .52 | .01 |
|  | Working memory | 247.66 | < .001 | .87 |  | 0.77 | .39 | .02 |  | 1.98 | .17 | .05 |
|  | Inhibition | 105.55 | < .001 | .74 |  | 0.08 | .79 | .002 |  | 0.53 | .47 | .01 |
|  | Cognitive flexibility | - | - | - |  | - | - | - |  | - | - | - |
|  | Composite score | 197.83 | < .001 | .84 |  | 1.13 | .30 | .03 |  | 1.53 | .22 | .04 |
| Distal outcomes – writing tasks | |  |  |  |  |  |  |  |  |  |  |  |
|  | Handwriting fluency | 18.08 | < .001 | .23 |  | 0.07 | .79 | .001 |  | 5.85 | .02 | .09 |
|  | Spelling accuracy | 97.65 | < .001 | .61 |  | 0.14 | .72 | .002 |  | 0.05 | .83 | .001 |
|  | Text quality | 14.72 | < .001 | .19 |  | 0.42 | .52 | .01 |  | 1.74 | .19 | .03 |
| Distal outcomes – teacher based | |  |  |  |  |  |  |  |  |  |  |  |
|  | Portuguese grades | 67.97 | < .001 | .52 |  | 0.76 | .39 | .01 |  | 4.39 | .04 | .07 |
